# Pre-Clinical Study of Panobinostat in Xenograft and Genetically Engineered Murine Diffuse Intrinsic Pontine Glioma Models

**DOI:** 10.1371/journal.pone.0169485

**Published:** 2017-01-04

**Authors:** Tammy Hennika, Guo Hu, Nagore G. Olaciregui, Kelly L. Barton, Anahid Ehteda, Arjanna Chitranjan, Cecilia Chang, Andrew J. Gifford, Maria Tsoli, David S. Ziegler, Angel M. Carcaboso, Oren J. Becher

**Affiliations:** 1 Department of Pediatrics, Duke University Medical Center, Durham, NC, United States of America; 2 Preston Robert Tisch Brain Tumor Center, Duke University Medical Center, Durham, NC, United States of America; 3 Preclinical Therapeutics and Drug Delivery Research Program, Fundacio Sant Joan de Deu, Barcelona, Spain; 4 Department of Pediatric Hematology and Oncology, Hospital Sant Joan de Deu, Barcelona, Spain; 5 Children’s Cancer Institute, University of New South Wales, Randwick, NSW, Australia; 6 Department of Anatomical Pathology, Prince of Wales Hospital, Randwick, NSW, Australia; 7 Kids Cancer Centre, Sydney Children’s Hospital, Randwick, NSW, Australia; 8 Department of Pathology, Duke University Medical Center, Durham, NC, United States of America; University of Michigan Medical School, UNITED STATES

## Abstract

**Background:**

Diffuse intrinsic pontine glioma (DIPG), or high-grade brainstem glioma (BSG), is one of the major causes of brain tumor-related deaths in children. Its prognosis has remained poor despite numerous efforts to improve survival. Panobinostat, a histone deacetylase inhibitor, is a targeted agent that has recently shown pre-clinical efficacy and entered a phase I clinical trial for the treatment of children with recurrent or progressive DIPG.

**Methods:**

A collaborative pre-clinical study was conducted using both a genetic BSG mouse model driven by PDGF-B signaling, p53 loss, and ectopic H3.3-K27M or H3.3-WT expression and an H3.3-K27M orthotopic DIPG xenograft model to confirm and extend previously published findings regarding the efficacy of panobinostat *in vitro* and *in vivo*.

**Results:**

*In vitro*, panobinostat potently inhibited cell proliferation, viability, and clonogenicity and induced apoptosis of human and murine DIPG cells. *In vivo* analyses of tissue after short-term systemic administration of panobinostat to genetically engineered tumor-bearing mice indicated that the drug reached brainstem tumor tissue to a greater extent than normal brain tissue, reduced proliferation of tumor cells and increased levels of H3 acetylation, demonstrating target inhibition. Extended consecutive daily treatment of both genetic and orthotopic xenograft models with 10 or 20 mg/kg panobinostat consistently led to significant toxicity. Reduced, well-tolerated doses of panobinostat, however, did not prolong overall survival compared to vehicle-treated mice.

**Conclusion:**

Our collaborative pre-clinical study confirms that panobinostat is an effective targeted agent against DIPG human and murine tumor cells *in vitro* and in short-term *in vivo* efficacy studies in mice but does not significantly impact survival of mice bearing H3.3-K27M-mutant tumors. We suggest this may be due to toxicity associated with systemic administration of panobinostat that necessitated dose de-escalation.

## Introduction

Diffuse intrinsic pontine glioma (DIPG) is a lethal, high-grade brainstem glioma (BSG) that originates in the pons, predominately in children. Despite numerous efforts to improve treatment, prognosis remains poor, with more than 90% of children dying within 2 years of diagnosis, making it one of the major causes of brain cancer-related deaths in childhood [1–3]. As surgical resection is not possible because of the tumor’s anatomic location, radiation therapy remains the only treatment with proven but temporary benefit, and no chemotherapy has shown efficacy over radiation alone [1, 4]. Genomic analysis of DIPG tissue obtained both at diagnosis and postmortem has unraveled the genomic landscape of the disease by identifying novel drivers of DIPG pathogenesis [5–11]. In particular, studies have identified highly recurrent mutations in genes encoding the histone variants H3.3 (*H3F3A*) and H3.1 (*HIST1H3B* or *HIST1H3C*) in approximately 80% of human DIPGs (as well as other midline gliomas) and to a lesser extent H3.2 (*HIST2H3C*), which results in broad epigenetic dysregulation [5, 6, 12–18]. These mutations produce an amino acid substitution conferring a change in lysine to methionine at position 27 on the histone tail (K27M) [5, 6, 12], which alters the distribution of the repressive trimethylation mark on H3K27 residues (H3K27me3) throughout the genome including a global loss leading to transcriptional de-repression [14–16]. Importantly, the H3.3-K27M mutation, which occurs in approximately 60% of human DIPGs, is associated with a proneural/oligodendroglial phenotype, amplifications/mutations of *PDGFRA*, *TP53* mutations, and a more aggressive clinical course than the H3.1-K27M mutation [17].

Histone deacetylases (HDACs) regulate the acetylation of histones in nucleosomes, which mediates changes in chromatin conformation, leading to regulation of gene expression. HDACs also regulate the acetylation status of a variety of other non-histone substrates, including key tumor suppressor proteins and oncogenes. Altered expression, downregulation, and mutations of HDAC genes are linked to tumor development. Histone deacetylase inhibitors (HDACis) are anti-proliferative agents that modulate acetylation by targeting histone deacetylases [19]. Panobinostat is a potent pan-histone deacetylase inhibitor of classes I, II, and IV that induces hyperacetylation of histones and other intracellular proteins, allowing for the expression of otherwise repressed genes, the inhibition of cellular proliferation and the induction of apoptosis in malignant cells [20]. In February 2014, the U.S. Food and Drug Administration approved panobinostat (Farydak) for the treatment of patients with multiple myeloma. In addition, panobinostat is used in several clinical trials for the treatment of various cancers including, but not limited to, leukemia, lymphoma, neuroendocrine tumors, renal cell cancer, non-small cell lung cancer, breast cancer, prostate cancer, colorectal cancer, and thyroid cancer. For adult brain tumors, NCT01324635 (clinicaltrials.gov) is an active phase I clinical trial investigating the combination of panobinostat and stereotactic radiation therapy.

In a recent pre-clinical study, Grasso, *et al*. found that panobinostat demonstrated therapeutic efficacy against DIPG both *in vitro* and *in vivo* [21]. Specifically, the drug was shown to be effective against H3-WT and H3-K27M DIPG cells *in vitro*, although H3-K27M-mutant cells developed resistance to panobinostat within weeks of exposure to low doses of drug. Similarly, systemic panobinostat treatment of H3.3-K27M-mutant NOD-SCID patient-derived orthotopic xenografts only temporarily slowed tumor growth, with tumors resuming rapid *in vivo* growth after 4 weeks of treatment. In addition, panobinostat treatment was shown to significantly prolong survival of mice bearing H3 wild-type tumors [21]. These findings led to the initiation of NCT02717455 (clinaltrials.gov), a phase I clinical trial of panobinostat (LBH589) through the Pediatric Brain Tumor Consortium (PBTC) for the treatment of children with recurrent or progressive DIPG.

To replicate and confirm the findings of Grasso *et al*., we conducted a collaborative pre-clinical study encompassing three institutions using both a genetically engineered mouse model (GEMM) of BSG driven by H3.3-K27M expression, activation of PDGF signaling, and p53 deficiency as well as a DIPG orthotopic xenograft mouse model harboring the H3.3-K27M and ACVR1-R206H mutations. The former model has been previously shown to recapitulate H3.3-K27M-mutant human tumors in the global loss of H3K27me3 [14], thus preserving the purported primary mechanism underlying the mutation. Our goal was to extend the observations of Grasso *et al*. to ascertain the efficacy of panobinostat in additional genetically and histologically faithful DIPG models.

## Results

### Panobinostat shows efficacy against murine brainstem glioma cells *in vitro* irrespective of H3 status

**Fig 1:**
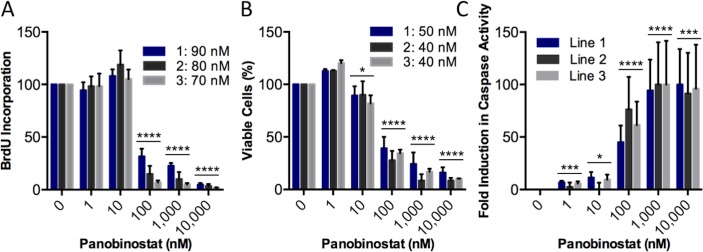
Panobinostat shows efficacy against brainstem glioma cells *in vitro*. **(A-C)** Mouse brainstem glioma cells driven by PDGF-B, H3.3-K27M, and Cre-induced p53 loss from three separate tumors were cultured as neurospheres and treated with varying doses of panobinostat for 48 h. (A) Proliferation assessment using a BrdU assay (**** p < 0.0001). (B) Cell viability assessment using a Celltiter-Glo assay (* p = 0.0252, **** p < 0.0001). (C) Apoptosis assessment using a Caspase-Glo 3/7 assay (* p = 0.0149, *** p = 0.0005, **** p < 0.0001). Values from each drug concentration were normalized to the control (0 nM panobinostat, 0.1% DMSO). IC50 values for each tumor cell line are shown in the legends. Each experiment was performed in triplicate and independently repeated three times for each tumor cell line. For all panels, error bars represent mean with SEM. Statistical significance to compare drug concentration groups to controls were determined using an unpaired two-tailed t-test.

To determine the effects of panobinostat on the growth, survival, and death of brainstem glioma (BSG) cells, we generated H3.3-K27M-expressing tumors by injecting neonatal Nestin-tv-a (Ntv-a);p53-fl/fl mice with RCAS-PDGF-B, RCAS-H3.3-K27M, and RCAS-Cre viruses as described in [22] and in Materials and Methods. In this autochthonous model, tumors are initiated via *in vivo* viral transduction of endogenous Nestin-expressing progenitors of the neonatal mouse brainstem, and tumor symptoms develop within 3–5 weeks of virus injection. We isolated three separate tumors, cultured the cells as neurospheres in stem/progenitor cell conditions and treated them with varying doses of panobinostat for 48 h. Cells were then assessed for proliferation, viability, and apoptosis. We found that panobinostat significantly reduced cell proliferation compared to the control as shown in Fig 1A. Likewise, we found that panobinostat significantly reduced cell viability compared to control as shown in Fig 1B. Having observed that panobinostat reduced the proliferation and viability of three tumor cell lines, we next investigated the effects of panobinostat on apoptosis and found that the drug significantly increased apoptosis compared to control (Fig 1C**)**, albeit to varying degrees among the different lines. Together, these findings suggest that panobinostat is a potent drug *in vitro* against murine brainstem glioma cells, confirming the findings of Grasso *et al*. regarding human DIPG cells [21].

**Fig 2:**
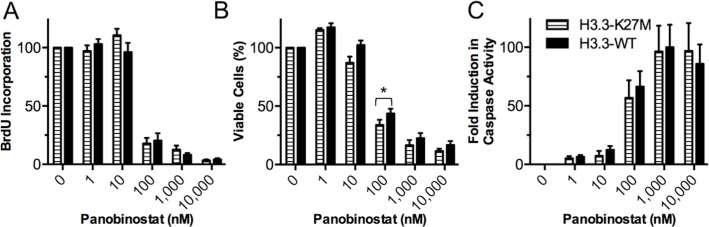
Panobinostat decreases proliferation and viability and induces apoptosis independent of H3.3-K27M status *in vitro*. Three independent murine brainstem glioma H3.3-K27M and H3.3-WT cell lines (driven by PDGF-B, Cre-induced p53 loss, and either H3.3-K27M or H3.3-WT, respectively) were treated with varying doses of panobinostat for 48 h. (**A**) Proliferation assessment using a BrdU assay. (**B**) Cell viability assessment using a CellTiter-Glo assay. (**C**) Apoptosis assessment using a Caspase-Glo 3/7 assay. Results from the different drug concentrations were normalized to the control (0 nM Panobinostat, 0.1% DMSO). Each experiment was performed in triplicate and independently repeated three times for each tumor cell line. Error bars represent mean with SEM. Statistical significance to compare H3.3-K27M and H3.3-WT cells at each drug concentration were determined using an unpaired two-tailed t-test (* p = 0.035).

Next, to investigate whether the cytotoxic effects of panobinostat are specific to BSG cells harboring the H3.3-K27M mutation, PDGF-B;p53-deficient murine tumors were generated that expressed either H3.3-K27M or H3.3-WT. Tumors were harvested, and cells were treated with varying doses of panobinostat for 48 h *in vitro*. The tumor cells were then assessed for proliferation, viability, and apoptosis. The results from three H3.3-K27M and three H3.3-WT tumor lines showed no statistically significant differences in cell proliferation, viability, or apoptosis following panobinostat treatment between the H3.3-K27M and H3.3-WT tumor lines, with the exception of a preferential inhibition of H3.3-K27M-mutant cell viability at the 10 nM dose (Fig 2). Thus, it can be concluded that in murine tumor cell lines, panobinostat is an effective drug independent of H3.3 status.

### Panobinostat potently reduces survival and clonogenicity of human patient-derived DIPG cells

**Fig 3:**
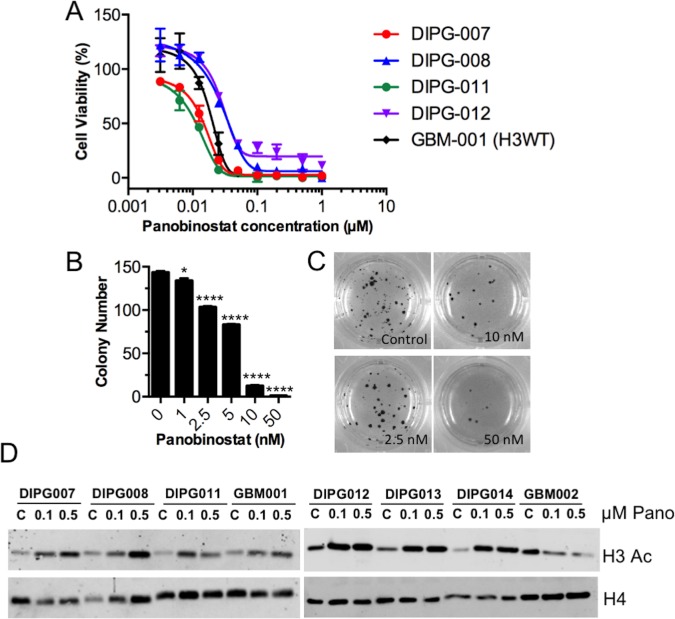
**Panobinostat inhibits survival and clonogenicity of human DIPG cells *in vitro*** (**A**) Human glioma cell models were treated with panobinostat for 72 h and then assayed for cell survival with an MTS Assay. Data are presented as the mean (with SD) of percent survival of control (untreated) cells. For each model, n = 6 replicates. (**B-C)** Human DIPG cells (HSJD-DIPG-007) were incubated with the indicated concentrations of panobinostat in soft agar for 2 weeks, and then colonies were stained with MTT and counted. Data in **B** are the mean with SEM, and statistical significance to compare drug concentration groups to controls were determined using an unpaired two-tailed t-test. * p = 0.02, **** p < 0.0001. Representative pictures of colonies are shown in **C**. (**D**) The indicated human glioma cell models were treated with either 0, 0.1, or 0.5 μM panobinostat for 48 h and then harvested for histone extraction. Histone lysates were separated via SDS-PAGE and blotted for the indicated antibodies.

Next, the impact of panobinostat treatment on the growth and clonogenicity of human glioma cells, including the H3.3-K27M-mutant DIPG model HSJD-DIPG-007 that harbors H3.3-K27M and ACVR1-R206H mutations [11], was investigated. It was found that panobinostat treatment reduces the survival (as measured by MTS assay) of a panel of human H3.3-K27M-mutant DIPG models, to a similar extent as an H3.3-WT cell model, with IC50s ranging from 12–28 nM (Fig 3A and Table 1). Panobinostat treatment, in addition, significantly reduced the clonogenicity of HSJD-DIPG-007 cells *in vitro* (Fig 3B and 3C) at low nanomolar concentrations.

**Table 1:** IC50s of human DIPG cell models with Panobinostat treatment.

Cell model	IC50 (nM)	95% CI
HSJD-DIPG-007	15.3	14.6–15.9
HSJD-DIPG-008	28.3	26.0–30.8
HSJD-DIPG-011	12.1	11.1–13.3
HSJD-DIPG-012	26.8	25.3–28.4
HSJD-GBM-001	18.0	16.6–19.6

Given that panobinostat is an inhibitor of histone deacetylases (HDACs), which remove acetyl groups from the lysine residues on histone tails, and as Grasso *et al*. showed increased H3 acetylation (H3Ac) with panobinostat treatment *in vitro* [21], we would expect the drug to increase the levels of histone acetylation within the DIPG cells used here. As shown by western blot (Fig 3D; for full blots see S1 Fig), panobinostat treatment at 0.1 and 0.5 μM for 48 h dose-dependently increased the H3 acetylation (H3Ac) levels in 6 different human H3.3-K27M mutant DIPG lines. Although the same was evident for the H3-WT model HSJD-GBM-001, this observation did not hold true for the H3.3-G34R mutant model HSJD-GBM-002, in which H3Ac levels actually decreased with panobinostat treatment.

### Pharmacokinetic studies reveal delivery of panobinostat into brain tissue, greater in the brainstem tumor than in the normal cerebral cortex *in vivo*.

**Fig 4:**
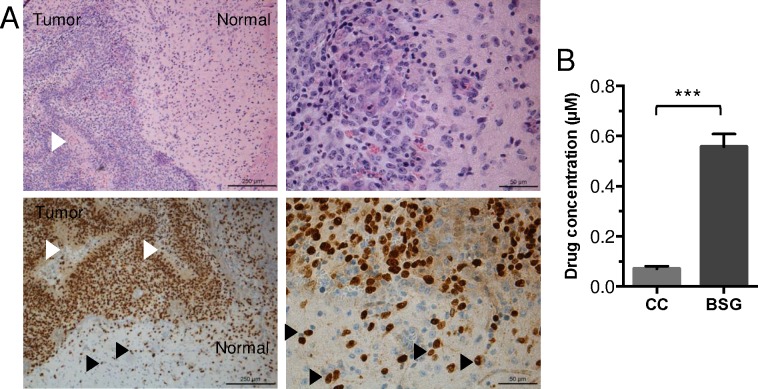
Pharmacokinetic studies reveal *in vivo* delivery of panobinostat into brain tissue of a murine brainstem glioma model, greater in the brainstem tumor than in the normal cerebral cortex. Neonatal Ntv-a;p53-fl/fl mice were injected with RCAS-PDGF-B, -H3.3-K27M, and -Cre viruses to induce tumor formation. (**A**) H&E (top panels) and IHC staining for HA (lower panels, brown nuclei, to identify the HA tag on the RCAS-H3.3-K27M construct) in tumors arising in these mice. Left panels, 100x, scale bar = 250 μm; right panels, 400x, scale bar = 50 μm). Note increased cellular density, pseudopalisading necrosis (white arrow heads), and invasion of HA+ tumor cells into normal brain (black arrow heads). (**B**) Upon the appearance of brain tumor symptoms (3–5 weeks post-injection), mice were treated with three doses of 20 mg/kg panobinostat (vehicle, 25% DMSO, 0.25x PBS, 5% glucose, n = 3) once daily by i.p. injections. Mice were then sacrificed 4 h after the final treatment. Pharmacokinetic analysis was performed to determine the concentration of panobinostat in the normal cerebral cortex and brainstem glioma tissue. Statistical significance was determined using unpaired two-tailed t-test to compare groups. CC = normal cerebral cortex, BSG = brainstem glioma.

One of the key obstacles to the treatment of DIPG is the delivery of therapeutic agents across the blood brain barrier (BBB). To determine if systemically administered panobinostat is able to cross the BBB and hence potentially target brain tumors, we investigated the pharmacokinetics of panobinostat in brain tissue *in vivo*. To test this, we used our autochthonous PDGF-B;H3.3-K27M;p53-deficient BSG GEMM, generated as described above by injecting RCAS-PDGF-B, -H3.3-K27M, and–Cre into Ntv-a;p53-fl/fl mice. These mice develop symptoms of brain tumors within 3 and 5 weeks post-virus injection, and resulting tumors recapitulate the high-grade and invasive features of human DIPG (Fig 4A). Upon the first appearance of brain tumor symptoms, a cohort of these mice were treated with three doses of 20 mg/kg panobinostat once daily by intraperitoneal (i.p.) injections. Mice were then sacrificed 4 h after the final treatment. Pharmacokinetic analysis was performed on tissue from the normal cerebral cortex and brainstem tumors. Our findings show that panobinostat was successfully delivered into both normal cerebral cortex and brainstem tumors (Fig 4B). In addition, panobinostat levels were significantly higher in brainstem tumor tissue as compared to cerebral cortex tissue of mice treated with drug (Fig 4B, 550±51 nM vs. 70±10 nM, mean±SEM, *** p = 0.0007). This suggests that in this BSG GEMM, the structural and functional integrity of the BBB is compromised to a greater extent in the tumors than in the normal cerebral cortex tissue of tumor-bearing mice. These results provide evidence that systemic delivery of panobinostat allows for drug concentrations in the brainstem that, according to our *in vitro* data, are above potentially active concentrations, precisely targeting the tumor tissue.

### Short-term *in vivo* treatments with panobinostat reduces tumor cell proliferation and increases H3 acetylation

**Fig 5:**
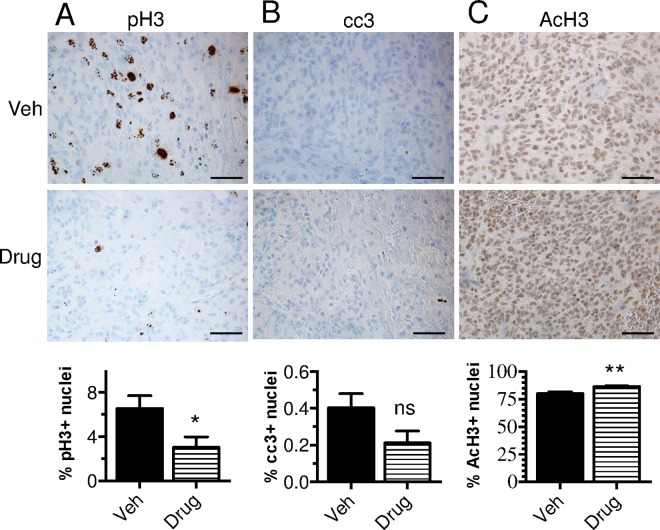
Short-term *in vivo* treatments with panobinostat reduces tumor cell proliferation and increases H3 acetylation without evidence of apoptosis. Neonatal Ntv-a;p53-fl/fl mice were injected with RCAS-PDGF-B, -H3.3-K27M, and–Cre viruses to induce tumor formation. Upon the first appearance of brain tumor symptoms (3–5 weeks post-injection), mice were treated with five doses of 20 mg/kg panobinostat (Drug) or vehicle (Veh, 25% DMSO, 0.25x PBS, 5% glucose, n = 6 in each group) once daily via i.p. injections and then sacrificed 1 hour after the final treatment. Shown is immunohistochemistry of brain tumor tissue for cell proliferation (phospho histone H3, pH3) **(A)**, cell apoptosis (cleaved caspase-3, cc3) (**B**), and H3 acetylation (AcH3) **(C**), 400x, scale bar = 50 μm, with quantification of total positive nuclear staining as a percentage of total nuclear area below in each panel. Statistical significance was determined using unpaired two-tailed t-test to compare groups. (ns: nonsignificant).

Given the evidence that panobinostat reaches the brainstem in our BSG GEMM and is cytotoxic against tumor cells *in vitro*, we investigated its short-term *in vivo* efficacy. To test this, murine PDGF-B; H3.3-K27M; p53-deficient brainstem tumors were induced as described above, and upon the first appearance of brain tumor symptoms, mice were treated with three doses of 10mg/kg panobinostat or vehicle once daily via i.p. injections and then sacrificed 1 hour after the final treatment (n = 5 in each group). Immunohistochemistry (IHC) staining of the brain tumor tissue for phospho histone H3 (pH3) and cleaved caspace-3 (cc3) showed no reduction in cell proliferation and no induction of apoptosis (data not shown). Subsequently, due to the possibility that the treatment regimen may have been insufficient to induce a therapeutic effect, the overall dose was increased, and mice received five doses of 20 mg/kg or vehicle (n = 6 in each group) once daily via i.p. injections and then were sacrificed 1 hour after the final treatment. Of note, the panobinostat treatment group initially consisted of 8 mice, however 2 of the mice were unable to tolerate all 5 doses, most likely due to drug toxicity, as discussed below. IHC of the brain tumor tissue for pH3 revealed a significant decrease in proliferation (* p = 0.0388, Fig 5A), however staining for cc3 did not detect an induction of apoptosis at this particular time point (p = 0.0883, Fig 5B).

Next, to investigate *in vivo* target inhibition, we performed IHC staining for AcH3 in brain tumor tissues collected from short-term treatment *in vivo* studies (symptomatic tumor mice treated with five doses of 20 mg/kg panobinostat or vehicle once daily via i.p. injections). As seen in Fig 5C, our results show a statistically significant, albeit small, increase in H3 acetylation in brain tumor tissue from mice treated with panobinostat as compared to vehicle (** p = 0.0057), thus supporting the drug’s proposed mechanism of action of HDAC inhibition.

### Panobinostat treatment *in vivo* does not induce an overall survival benefit

**Fig 6:**
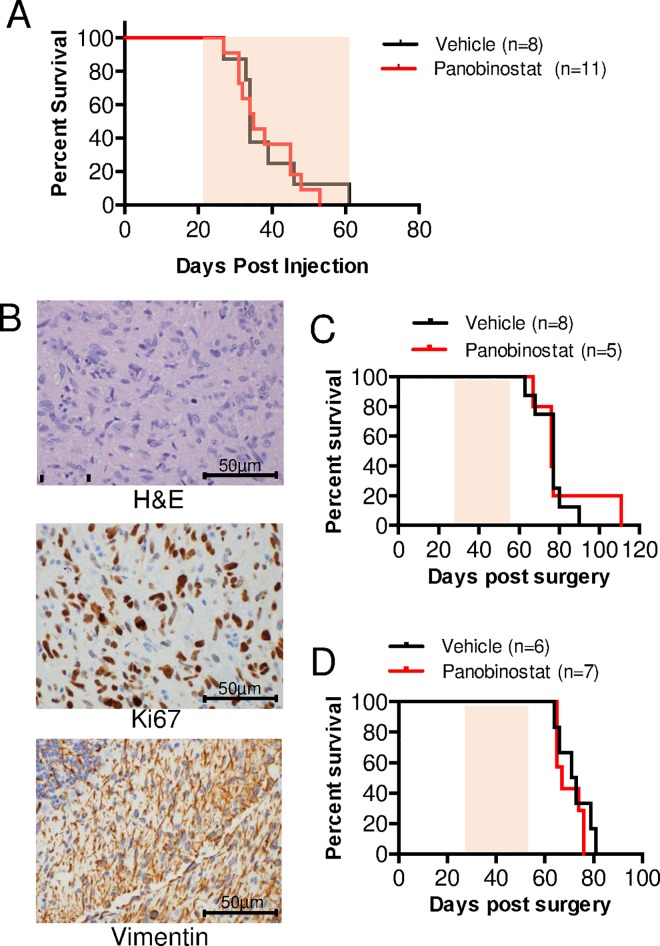
No overall survival benefit with panobinostat treatments *in vivo*. **(A)** Mice with tumors driven by PDGF-B and p53 loss harboring H3.3-K27M mutations (generated as described in Materials and Methods) were treated with 20 mg/kg panobinostat (n = 11) or vehicle (25% DMSO, 0.25x PBS, 5% glucose, n = 8) administered via intraperitoneal (i.p.) injection once per day twice a week beginning 21 days post-virus injection and continuing until mice reached humane endpoints. The mice were monitored daily and sacrificed upon moribund condition (lethargy, enlarged head circumference, ataxia, and/or > 25% weight loss) (p = 0.756, log rank test). **(B-D)** Effect of panobinostat treatment on the survival of mice-bearing H3.3-K27M HSJD-DIPG-007 orthotopic xenografts. NOD-SCID mice (7 weeks old) were orthotopically injected with HSJD-DIPG-007 cells (passage 39) into the brainstem via stereotactic coordinates. (**B**) Representative H&E (top panel) and IHC staining for Ki67 (middle panel) and Vimentin (bottom panel) of control mice (treated with vehicle and sacrificed immediately after the last dose, as described in the Materials and Methods). 400x magnification, scale bar = 50 μm. (**C-D**) Starting on day 28 post-implantation, mice were treated with panobinostat prepared in a vehicle containing 5% dextrose (**C**) or 2.5% DMSO, 5% PEG400 and 5% Tween80 in 0.9% saline (**D**) via intraperitoneal (i.p.) injection at 10 mg/kg, three times a week for four weeks (*p*>0.05, log-rank test). Shaded areas under the curves in **A, C-D** indicate treatment duration.

We next investigated whether the significant decrease in tumor cell proliferation after treatment with panobinostat, as shown in both *in vitro* experiments and short-term *in vivo* treatments, would translate into an overall survival benefit in H3.3K27M-mutant models. To test this, we first used our PDGF-B;H3.3-K27M;p53-deficient BSG GEMM. Mice were initially treated once daily with 20 mg/kg drug or vehicle via i.p injections beginning 21 days post virus-injection. However, apparent toxicity at this dose necessitated dose de-escalation to 10 mg/kg once daily, then 10 mg/kg every other day, followed by 20 mg/kg once per week. Panobinostat at 20 mg/kg once per week was well-tolerated, however there was no overall survival benefit in the panobinostat-treated mice in comparison to the vehicle-treated mice (S2A Fig). Subsequently, due to the possibility that the lack of a survival benefit was secondary to an insufficient dose, the dose was increased to 20 mg/kg once daily administered twice per week. This dose was also well-tolerated, however our results show no overall survival benefit in the panobinostat-treated mice in comparison to the vehicle-treated mice (Fig 6A, median survival 35 days versus 34 days, respectively). Tumors in the brainstem were confirmed in all mice by hematoxylin and eosin (H&E) staining. The treatment dose, frequency, and/or duration was not further increased due to the high likelihood of panobinostat toxicity. Thus, at the well-tolerated dose of 20 mg/kg administered once daily twice per week, panobinostat did not prolong overall survival of our BSG GEMM.

Next, the efficacy of panobinostat in a human DIPG orthotopic xenograft model was assessed. HSJD-DIPG-007 cells were orthotopically implanted into the pons of 7-week old NOD SCID mice using stereotactic coordinates. In this model, mice exhibit symptoms of brain tumor formation within 60–80 days post tumor cell implantation and harbor tumors that histologically resemble human DIPG, with tumor cells infiltrating the brainstem as seen with routine hematoxylin and eosin staining and the cerebellum as shown by vimentin staining (Fig 6B). Beginning on day 28 post-implantation, mice were treated with 10 mg/kg panobinostat or vehicle (5% dextrose) once daily by i.p. injection. All mice exhibited significant toxicity following the second dose of panobinostat, indicated by decreased motor activity, ruffled fur, hunched posture, labored breathing, average weight loss of 18%, and death. Autopsy of the deceased animals revealed a yellow secretion in the stomach cavity. Due to the severe toxicity, the experiment was continued with the administration of panobinostat on alternating days (3 days per week) for 4 weeks. With this regimen, no severe toxicity was observed. However, mice suffered from diarrhea and were sustained with mushy food, jelly and 5% i.p. glucose daily for the duration of the experiment. As shown in Fig 6C, no survival difference was observed between vehicle-treated control and panobinostat-treated mice (*P* >0.05, log-rank analysis).

As panobinostat is insoluble in 5% dextrose, it was next solubilized in 2.5% DMSO, 5% PEG400 and 5% Tween80 in 0.9% saline. Mice harboring intracranial xenografts were treated with once daily 10 mg/kg panobinostat three times per week (M,W,F) for four weeks starting at 28 days post-implantation. Using this vehicle and treatment regimen, no signs of toxicity were observed in panobinostat-treated mice. However, no survival difference was observed between mice treated with panobinostat and those treated with vehicle (Fig 6D, *P* >0.05, log-rank analysis).

Finally, an alternative vehicle and dosing regimen was tested, with mice harboring intracranial HSJD-DIPG-007 xenografts receiving either no treatment or 10 mg/kg panobinostat [in 10% DMSO diluted in PBS-10% hydroxypropyl-beta-cyclodextrin (HPBCD)] once daily via i.p. injection starting at day 23 post tumor cell implantation for 5 days on followed by 2 days off and then 5 days on again, for a total of 10 doses (S2B and S2C Fig). This dosing regimen aimed to reproduce the regimen previously reported by Grasso *et al*. to temporarily slow H3-K27M tumor growth and increase survival in an H3-WT DIPG model [21]. However, there was no survival benefit observed for the H3.3-K27M DIPG model used here with this drug treatment (median survival of 81.5 and 80 days for treated and control mice, respectively).

## Materials and Methods

### Duke University Medical Center, USA

#### Mice

All *in vivo* experiments associated with Figs 1, 2, 4, 5, 6A and S2A were performed in accordance with the Duke University Animal Care and Use Committee and Guide for the Care and Use of Laboratory Animals (protocol # A214-13-08). Nestin-Tv-a (Ntv-a); p53^fl/fl^ mice have been previously described [22] and were created by breeding Ntv-a mice with p53-floxed mice (C57BL/6J background) from Jackson Labs.

#### Autochthonous brainstem glioma GEMM

To generate viruses for the induction of brainstem gliomas (BSGs) in mice, DF1 virus-producing cells were purchased from ATCC, cultured in DMEM (ATCC) supplemented with 10% FBS, 2mM L-glutamine, 100 units/mL penicillin and 100 μg/mL streptomycin and incubated at 39°C and 5% CO_2._ Cells were transfected with RCAS plasmids (RCAS-PDGF-B, RCAS-Cre, RCAS-H3.3K27M, or RCAS-H3.3WT) using Fugene 6 or X-TremeGENE 9 (Roche) per the manufacturer’s instructions. Virus-producing cells were used for injections after being passaged at least 6 times and less than 20 times from the time of transfection. To generate BSGs, Nestin-tv-a(Ntv-a); p53^fl/fl^ mice (that express tv-a, the receptor for RCAS viruses, in Nestin-expressing cells) were injected with 1 μL of a 1:1:1 cocktail of DF1 cells expressing RCAS-PDGF-B, RCAS-Cre, and RCAS-H3.3-K27M (or RCAS-H3.3-WT) as previously described [23]. Injections were made 2 mm posterior to the bregma along the midline using a Hamilton syringe and custom needle. Injections were performed on postnatal day 2 to postnatal day 5 mice after being anesthetized on ice. Mice were monitored daily and euthanized with CO_2_ upon the appearance of signs of brain tumors (lethargy, enlarged head, ataxia, weight loss up to 25%).

#### Generation of murine BSG neurospheres

To generate BSG cell lines, tumors were isolated from symptomatic mice (described above) and enzymatically digested in Earl’s Balanced salt solution containing 4.7mg papain (Worthington) and 60μg /mL DNAse (Sigma Aldrich). Digestion was inactivated with ovomucoid (0.7 mg/mL) (Worthington) containing 14μg/mL DNAse. Cells were consecutively washed, triturated, and strained to obtain a single cell suspension. The cells were cultured in neurosphere media [Neurocult media (Stem Cell Technologies) supplemented with 5 ml cell proliferation supplement (Stem Cell Technologies), 500 μL penicillin and streptomycin, 10 μL human basic FGF (20 ng/ml), 5 μL human EGF (10 ng/ml), 50 μL heparin] and incubated at 37°C and 5% CO_2._

### *In vitro* assays on murine BSG cells

#### Cell proliferation

Neurospheres generated from BSGs were plated as single cells in triplicate in a white-walled, clear-bottomed 96-well plate (50,000 cells/well in 100 μL) and allowed to adhere for 24 h. Cells were then treated with 10 μL working solution [1 μL of panobinostat (Selleckchem, Catalog #S1030) stock solution (serial dilutions of 10 mM) + 99 μL neurosphere media (described above)] at increasing panobinostat concentrations (0.001 μM, 0.01 μM, 0.1 μM, 1.0 μM, 10 μM) or 0.1% DMSO (control) for 48 h. A bromodeoxyuridine (BrdU) based cell proliferation ELISA assay kit (Roche) was used to assay proliferation. Cells were pulsed with BrdU for 4 h. Absorbance was read using Molecular Devices Versa Max Tunable Microplate Reader. Normalized proliferation levels were calculated relative to the vehicle (0 μL panobinostat). The dose response curve was generated with Prism software and analyzed using nonlinear regression. The values of half-inhibitory concentration (IC50) were calculated by using log (agonist) versus response including variable slope (four parameters) statistics and normalized in GraphPad Prism. Each experiment was performed in triplicate and independently repeated three times for each tumor cell line. Error bars represent mean with SEM. Statistical significance to compare drug concentration groups with controls were determined using an unpaired two-tailed t-test.

#### Cell viability and apoptosis

BSG neurospheres were generated, cultured, and treated as above for cell proliferation. For cell viability, the CellTiter-Glo Luminescent Assay (Promega) was performed, and for apoptosis, the Promega ApoTox-Glo Triplex Assay (# G6321) was used, both according to the manufacturer’s instructions. Luminescence was read using a Turner Biosystems Modulus Microplate Reader. Data analysis and statistics were performed as above for cell proliferation.

### Short-term panobinostat treatment of BSG GEMM

Upon the first appearance of brain tumor symptoms, tumor-bearing mice were treated with either 1) three doses of panobinostat (Selleckchem #S1030) at 10 mg/kg or vehicle (25% DMSO, 0.25x PBS, 5% glucose) administered once daily by i.p. injections (n = 5 in each group), or 2) five doses of panobinostat (Selleckchem) at 20 mg/kg or vehicle administered once daily by i.p. injections (n = 6 in each group). Mice were sacrificed 1 hour after their final treatment via CO_2_, and their brains were extracted, fixed in 10% formalin, paraffin embedded and sectioned on a microtome. Immunohistochemistry for phospho histone H3 (pH3), cleaved caspase-3 (cc3), and H3 acetylation (AcH3) were conducted with subsequent quantification (described below).

### Immunohistochemistry

Formalin fixed brains were paraffin embedded by Duke Pathology Core Services. Sections were cut 5μm thick using a Leica RM2235 Microtome. Digital images of microscopic fields of brain tissue sections were acquired with a Leica DMLB microscope, Leica digital camera and Leica Application Suite Version 3.7 (Leica; Buffalo Grove, IL) at 400x magnification (high powered field). Hematoxylin and Eosin (H&E) staining was performed using standard protocols. Immunohistochemistry was performed using an automated processor (Discovery XT, Ventana Medical Systems, Inc.) Antibodies used were as follows: anti-HA (Y-11, 1:250 dilution, Santa Cruz #sc-805), anti-phospho-Histone H3 (Ser10) (1:1600 dilution, Millipore #04–1093), anti-cleaved caspase 3 (Asp175) (1:1600 dilution, Cell Signaling #9661), and anti-acetyl-Histone H3 (1:500 dilution, Millipore #06–599). Quantification analysis was performed blindly using MetaMorph Image Analysis Software. Percentage of positive staining cell nuclei equal to positive nuclear area (brown staining only with a specific pre-set threshold) / total nuclear area (blue and brown staining) x 100. Thresholds for positive nuclei and total nuclei were unchanged throughout the quantification process. Statistical significance was determined using unpaired two-tailed t-test to compare groups.

### Pharmacokinetics

Symptomatic mice (BSG GEMM) were treated with three doses of 20 mg/kg panobinostat (Selleckchem) or vehicle (25% DMSO, 0.25x PBS, 5% glucose) (n = 3 in each group) administered once daily by intraperitoneal injections. Mice were sacrificed 4 h after their final treatment via CO_2_. Brains from the sacrificed mice were extracted. Half of each brain was fixed in 10% formalin and embedded in paraffin for histological analysis. The other half was SNAP frozen, stored at -80 degrees and sent for pharmacokinetic (PK) studies. Pharmacokinetic analysis was performed on the cerebral cortex tissue and brainstem tumor tissue of each mouse by the Pharmacokinetic/Pharmacodynamic (PK/PD) Core Laboratory, Duke Cancer Institute as described below. Statistical significance was determined using unpaired two-tailed t-test to compare groups.

#### Tissue processing

Brain tissue was homogenized with 2 parts water (w/v) by rotary homogenizer (polyethylene rotor/1.5-mL conical tube). To a 200-μL PP tube, 20 μL of tissue homogenate and 40 μL of methanol (containing 10 ng/mL PSTAT-d8 internal std.) was added and vigorously agitated in a FastPrep vortexer (Thermo-Savant) at speed 4 for 20s. After centrifugation at 13,600 g for 5 min at RT, the supernatant was transferred into an injection vial, and 10 μL was injected into the LC/MS/MS system.

#### Liquid chromatography tandem-mass spectrometry (LC/MS/MS)

The analysis was performed on a Shimadzu 20A series LC system coupled with an Applied Biosciences/SCIEX API 4000 QTrap MS/MS spectrometer. Column: Phenomenex, C18 4x3 mm (P/N AJ0-4287) column at 35°C. Mobile phase solvents (all MS-grade): A—0.1% formic acid in water, 2% acetonitrile; B–acetonitrile. Elution gradient at 1 mL/min: 0–0.5 min 0–95% B, 0.5–1.0 min 95% B, 1.0–1.2min 95–0% B. Run time: 4min. MRM transitions for PSTAT and PSTAT-d8 (m/z): 350/158 and 358/164, respectively. Positive-ion mode. DP: 76 V, EP: 10 V, ion-spray voltage: 5500 V, curtain gas: 30, ion-source gas1: 30, ion-source gas2: 30. Lower limit of quantification (LLOQ): 1.2 ng/g wet tissue. Calibration curve samples (n = 6) were prepared by adding increasing amounts of Panobinostat to control brain homogenate obtained from non-treated animals.

### Long-Term panobinostat treatment of BSG GEMM

Tumor-bearing mice were randomly assigned to be treated with either 20 mg/kg panobinostat or vehicle (25% DMSO, 0.25x PBS, 5% glucose) once daily administered once or twice a week via i.p. injections. This dose was reduced from 20 mg/kg daily to 10 mg/kg daily and 10 mg/kg every other day due to toxicity concerns. Of note, 25% DMSO was the lowest percentage in which panobinostat could be successfully and reproducibly solubilized in our hands. Treatments began 21 days post-injection with RCAS-PDGF-B, RCAS-Cre, and RCAS-H3.3-K27M expressing cells and continued until mice reached humane endpoints. Mice were monitored daily and sacrificed upon moribund conditions (lethargy, enlarged head circumference, ataxia, and/or > 25% weight loss) or 12 weeks post-injection in the absence of symptoms. Statistical significance between the Kaplan-Meier survival curves was determined using a log-rank (Mantel-Cox) test.

### Children’s Cancer Institute, Australia

#### Cell lines and reagents

The pediatric autopsy-derived DIPG cell line HSJD-DIPG-007 (DIPG-007) was obtained from Dr. Angel M. Carcaboso (Hospital Sant Joan de Déu Barcelona, Barcelona, Spain; for details see below). Cells were cultured as neurospheres in tumor stem base medium (50:50 DMEM/F12:Neurobasal Life Technologies) supplemented with B-27 (Life technologies), Heparin (Stemcell Technologies) and human growth factors EGF, FGF-basic, PDGF-AA and PDGF-BB (Jomar Life Research). Cells tested free of mycoplasma contamination. Panobinostat was purchased from Selleckchem.

#### Clonogenic Assay

Clonogenic assay was performed using a soft agar method. Plates were coated with 0.5% SeaPlaque agarose (Cambrex) in tumor stem base medium and stored at 4°C overnight. The following day, 0.3% agarose containing HSJD-DIPG-007 cells and various concentrations of panobinostat were plated over the agar underlay. The plates were incubated at 37°C for two weeks. Colonies were then stained using MTT (3-[4,5-Dimethylthiazol-2-yl]-2,5-diphenyltetrazolium bromide; Thiazolyl blue) and counted.

#### Mice

All *in vivo* experiments were performed in accordance with the University of New South Wales Animal Care and Ethics Committee according to the Animal Research Act, 1985 (New South Wales, Australia) and the Australian Code of Practice for Care and Use of Animals for Scientific Purposes (2013). The animal studies associated with Fig 6B–6D were conducted according to the protocols approved by the Animal Experimentation Ethics Committee of the University of New South Wales (ACEC number: 16/7A). NOD/SCID mice (Fig 6B–6D) were obtained from Animal Resources Centre (ARC, Canning Vale Western Australia) and maintained in a temperature-controlled environment with a 12-hour light/dark cycle in Tecniplast individually vented cages with enviro-dri and igloos for nesting. Mice were given irradiated feeder pellets and water ad libitum. Mice were euthanized at the completion of the protocol by carbon dioxide overdose.

#### Orthotopic injection

To orthotopically implant tumor cells into 7 week old mice, stereotactic surgery was performed using a KOPF small animal stereotactic surgical device. Mice were sedated using isoflurane gas. The skull of each mouse was exposed by incising the skin, and a small burr hole was made using a high-speed drill. Exact coordinates that target the pons region via the IVth ventricle were used (0.5mm lateral to the sagittal suture, 5.4mm posterior to the bregma and 3.1mm deep). A total of 3x10^5^ HSJD-DIPG-007 cells (passage 39) suspended in 2 μL of matrigel were injected using a point style 2, 25 gauge Hamilton syringe. Upon sealing the surgery site, mice were given 10μg/kg Buprenorphine intraperitoneally. Mice were placed on a warming pad and returned to their cages upon full recovery.

#### Histological analysis of orthotopic xenografts

Mice bearing orthotopic xenografts generated as described above were treated with vehicle (both Tween/PEG and 5% dextrose via i.p. injection Monday, Wednesday and Friday for a total of 12 treatments and sacrificed 24 hours after the last dose. Mouse brains were harvested and following formalin fixation were sectioned in a mouse brain cutter into either coronal or sagittal sections. Processing of tissue was performed in the Histology Unit at the Garvan Institute of Medical Research. Morphologic assessment of xenografts is based on routine haematoxylin and eosin (H&E) staining and immunohistochemical (IHC) staining with Vimentin (1:400; Novocastra NCL-VIM-V9) and Ki67 (1:400; Thermoscientific; RM-9106S1). Representative photographs of a vehicle control xenograft (shown in Fig 6B) were taken using an Olympus BX53 light microscope and CD-73 camera with cellSens software.

#### Panobinostat drug preparation and treatment of orthotopic xenografts

Panobinostat (Selleckchem) was prepared in two different vehicles. For the first *in vivo* experiment (Fig 6C) a stock solution of 1 mg/ml of panobinostat was prepared in 5% dextrose, and for the second experiment (Fig 6D), panobinostat was dissolved in DMSO at the concentration of 40 mg/ml and then diluted with 0.9% saline containing 5% PEG400 and 5% Tween80. The final concentration of panobinostat in the solution was 1 mg/ml. The drug was prepared fresh before treatment. In experiment 1, mice were treated daily with 10 mg/kg panobinostat intraperitoneally, which was decreased to three times a week due to toxicity, for four weeks. For the experiment 2, the animals were treated with 10 mg/kg three times a week (M,W,F) for four weeks intraperitoneally. Control groups were administered with the drug-free vehicle. The treatment was started on day 28 after the tumor implantation. Mice were sacrificed following the development of any of the following clinical signs of tumor progression: severe head tilting, severe ataxia, severe circling, lethargy and/or weight loss ≥20% of the initial weight.

#### Statistical analysis

For survival analysis of orthotopic xenografts, log-rank analysis was used.

### Hospital Sant Joan de Déu Barcelona, Spain

#### Human DIPG cell culture

Patient-derived DIPG cell models were obtained under an Institutional Review Board-approved protocol and with written informed consent (M-1608-C) at Hospital Sant Joan de Deu Barcelona, Spain. Models were established either from patient autopsy (HSJD-DIPG-007; from a patient who survived less than one month after diagnosis and was treated with one cycle of irinotecan-cisplatin and no radiation therapy; original tumor sequenced in [11], coded HSJD_DIPG007, with H3.3-K27M and ACVR1-R206H mutations), or from biopsies at diagnosis (DIPG models HSJD-DIPG-008, HSJD-DIPG-011, HSJD-DIPG-012, HSJD-DIPG-013, HSJD-DIPG-014, all of which are H3.3-K27M-mutated and were established from pons biopsies; and HGG model HSJD-GBM-001, H3-WT, established from tumor in frontal lobe), or from biopsies at relapse (HGG model HSJD-GBM-002, H3.3-G34R-mutated, established from a tumor in the left hemisphere). Cells were cultured as tumorspheres as previously described in [24].

### *In vitro* experiments with human DIPG cells

#### MTS Assay

To study the antiproliferative activity of panobinostat on human DIPG and HGG cells, tumorspheres in culture were disaggregated either mechanically or with TrypLE Express Stable Trypsin Replacement Enzyme (Life Technologies), and 3,000 cells per well were plated in 96 well-plates and maintained in culture for 24 h. Cells were then treated with panobinostat (Seqchem, Pangbourne, UK; stock 5 mg/mL in DMSO) at concentrations ranging 0.003–1 μM for 72 h, six wells per condition. The final DMSO concentration was ≤ 0.014% for all conditions; previous data (not shown) indicates that up to 0.1% DMSO does not affect the growth of these DIPG models. Control cells were untreated. Cell viability was assessed by the MTS assay (Promega, Fitchburg, WI, USA), and IC50 values were calculated with Graphpad.

#### Immunoblotting

Tumor cell lines were lysed in EpiSeeker Histone Extraction Kit (Abcam). Each histone lysate (1 μg) was separated by SDS-PAGE and transferred onto a nitrocellulose membrane (Thermo Scientific). Membranes were blocked in Tris-buffered saline with 5% nonfat dry milk and incubated overnight at 4°C with rabbit anti-Histone 4 (1:1,000, Abcam, catalog number ab10158) and rabbit anti-histone H3.3, Acetylated (1:1,000, Millipore, catalog number: 06–599), followed by horseradish peroxidase-conjugated secondary antibodies (1:10,000, Promega).

### Panobinostat treatment of orthotopic xenografts in mice

The xenograft studies of panobinostat activity intended to reproduce the previously reported preclinical study using an H3-WT DIPG model [21]. NOD.SCID mice were obtained from Harlan (Barcelona, Spain). Animal studies associated with S2B and S2C Fig were performed according to the Institutional and European guidelines (EU Directive 2010/63/EU) and were approved by the local animal care and use committee (Comité Ético de Experimentación Animal at Universidad de Barcelona, protocol 135/11). To establish orthotopic tumors, 3-week old mice (5 per group) were anesthetized with 100 mg/kg ketamine and 10 mg/kg xylazine and immobilized in a stereotaxic apparatus (Stoelting, Wood Dale, IL). A burr hole was drilled in the skull at coordinates (from bregma) x+0.5 and y-5.4, and 5 x 10^5^ HSJD-DIPG-007 cells (p69) suspended in 5 μL matrigel (BD Biosciences) were injected at 3.1 mm depth (targeting the 4^th^ ventricle) with a dull 22G needle attached to a 50 μL syringe (Hamilton, Bonaduz, Switzerland), using a stereotaxic arm. Animals recovered and treatments were initiated 23 days after tumor cell implantation. Panobinostat was prepared each day from frozen stocks in DMSO. Final formulation was 10% DMSO diluted in PBS containing 10% hydroxypropyl-beta-cyclodextrin (HPBCD) as a solubilizer, for the regimen of 10 mg/kg 5 days on, 2 days off, 5 days on (S2B Fig). Control mice received no treatment. Tumor endpoints used were weight loss >20% from maximum weight achieved or severe motor symptoms. For survival analysis of orthotopic xenografts, log-rank analysis was used.

### Histological analysis of orthotopic xenografts

Tumors from orthotopic DIPG xenografts, generated as described above, were harvested when mice reached humane endpoints and formalin-fixed paraffin-embedded. Heat-induced antigen retrieval with sodium citrate was performed according to standard protocols using an indirect immunoperoxidase method. The primary antibodies used were mouse anti-human Nuclei (1:200, MAB4383, Merck Millipore), mouse anti-Ki67 (Bond ready to use reagent, Clone K2, Leica Biosystems), and mouse anti-Histone H3 trimethyl K27 (1:600, Millipore, catalog number: 07–449). Staining was visualized with Novolink Polymer Detection Systems from Leica, followed by hematoxylin counterstaining (S2C Fig**)**.

## Discussion

Despite numerous efforts to improve the treatment of DIPG the prognosis remains poor. Targeted therapy with panobinostat has recently entered a phase I clinical trial for the treatment of children with recurrent or progressive DIPG due to promising pre-clinical studies [21]. To replicate and extend these previously published findings, we conducted a cooperative pre-clinical study encompassing three institutions using both genetically engineered and orthotopic patient-derived DIPG mouse models to investigate the efficacy of panobinostat *in vitro* and *in vivo*.

*In vitro*, we confirmed panobinostat to be highly effective against DIPG tumor cells at low nanomolar to low micromolar concentrations, depending on the cell and assay type. In addition, we found the sensitivity of DIPG cells to panobinostat to be independent of the H3.3 mutational status, as both H3.3-K27M and H3.3-WT cells were equally susceptible to treatment. Combined with results reported by Grasso *et al*. who observed effects of panobinostat on H3-WT, H3.1-K27M, and H3.3-K27M DIPG cells [21], this confirms that panobinostat could be an option for the treatment of all DIPG subtypes, even when the H3.3-K27M mutation is not present or if its status is unknown.

Importantly, the study here advances the current literature on the use of panobinostat to treat DIPG by demonstrating histological evidence of target inhibition *in vivo*. The short-term treatment of BSG-bearing mice with panobinostat at 20 mg/kg once daily for 5 days resulted in a significant decrease in cell proliferation, however, we were unable to detect an induction in apoptosis via cleaved caspase 3 staining at the time point investigated. It is possible that an alternative (more sensitive) assay or time point could have revealed an induction of apoptosis; however the reduced proliferation may have been achieved by an alternate mechanism, such as epigenetic modifications. For instance, pharmacodynamics analysis showed increased levels of H3 acetylation in tumor tissue demonstrating target inhibition at this dosing schedule. Pharmacokinetic studies revealed successful delivery of panobinostat into brain tissue of mice bearing genetically engineered BSG, providing evidence that it is able to reach potentially active concentrations in tumor tissue in this model. Furthermore, the distribution of panobinostat was higher in these brainstem tumors as compared to the normal cerebral cortical tissue, likely due to a compromised structural integrity of the BBB, which has been previously demonstrated in our GEMM [25], thus allowing a greater concentration of panobinostat to penetrate, and potentially treat, the tumor. Whether panobinostat reached potentially active levels in the HSJD-DIPG-007 xenograft model was not addressed here.

Grasso *et al*. reported temporarily reduced H3.3-K27M tumor growth (via bioluminescence) with systemic panobinostat administration of 10 mg/kg 3 times per week to a NOD-SCID patient-derived orthotopic xenograft DIPG model, with tumor regrowth evident after 4 weeks of therapy; the overall survival of these mice was not reported [21]. In addition, they observed significantly prolonged survival with systemic panobinostat treatment (10 mg/kg 5 days on 5 days off) as compared to vehicle treated controls using an H3 wild-type DIPG model [21]. The integrity of the BBB in this xenograft model was not reported, although panobinostat concentrations were determined to be approximately 200 nM in the pons of nontumor mice [21]. In our studies, we confirm a short term effect on H3.3-K27M tumor cell growth (via reduced proliferation after 5 days of treatment) in a BSG GEMM and observe no survival benefit of systemic treatment with panobinostat as compared to vehicle treated controls with various dosing regimens in immunocompromised xenograft models and immunocompetent GEMMs. This lack of efficacy to improve animal survival is inconsistent with our data showing high drug distribution in the GEMM tumors (above 500 nM) and could be due to the reduced dosage required to avoid toxicity, the development of drug resistance as was shown previously *in vitro* [21], or, for the orthotopic xenograft model, the high passage number of cells transplanted, which may have selected for particularly aggressive subclones. Despite the late cell passage number of this model, however, it represents one of the few molecularly, histologically, and clinically faithful orthograft DIPG models available. When considered along with the results of Grasso *et al*., there may be a discrepancy in drug response *in vivo* between H3.3-K27M and H3-WT DIPG models, potentially a result of the mutation, although this will require further direct investigation to confirm. Although the *in vitro* studies reported here and previously showed that panobinostat was potently effective against all tumor cell lines independent of H3 status, a multitude of other factors contribute to overall survival in *in vivo* studies.

It is also important to note that based on the standard deviation of the BSG GEMM used here, in order for a 5 day-difference in median survival to be statistically significant (as was shown in a previously published study using an Ink4a-ARF deficient BSG GEMM to test the CDK4/6 inhibitor PD-033299 [23]), a minimum of 42 (for 90% power) or 32 (for 80% power) mice would need to be treated with drug. Therefore, our study reported here may have been insufficiently powered to detect a small survival benefit with systemic panobinostat treatment, although all data (including GEMM and orthotopic xenograft studies) are sufficiently powered to detect a clinically-meaningful 10–12 day difference in survival (with 80% or 90% power).

A couple noteworthy potential challenges in using panobinostat for the treatment of DIPG in children are drug toxicity and inadequate drug delivery. In the *in vivo* studies shown herein, the drug concentration and duration of panobinostat treatments required to achieve an overall survival benefit would have likely resulted in substantial toxicity. As it is not yet known whether this toxicity translates to children with DIPG, these results should be taken into consideration during the execution of clinical trials with panobinostat. In addition, although data with our GEMM suggest that the BBB structural integrity is likely compromised in these tumors, as evidenced by the increased drug concentration in the tumor tissue compared with normal tissue, this may not hold true for children with DIPGs treated with panobinostat. The lack of efficacy of systemic chemotherapy or targeted agents in DIPG has been partly attributed to their poor delivery into the tumor because of a relatively intact BBB, as evidenced by the minimal contrast enhancement in DIPGs with magnetic resonance imaging [3]. In fact, the BSG GEMM used here, although compromised relative to normal brain [25], exhibits reduced permeability as compared with tumors in the cerebral cortex [26]. One possible solution would be drug delivery via convection enhanced delivery (CED), a neurosurgical technique in which the drug is delivered through one to several catheters placed stereotactically directly within the tumor mass or around the tumor or resection cavity [27]. This technique would circumvent the BBB and deliver panobinostat directly into the brainstem tumor, possibly without the toxicity associated with systemic administration. Indeed, Grasso *et al*. reported significant reduction in H3.3-K27M orthotopic tumor growth after just one administration of panobinostat via CED [21].

In summary, our pre-clinical study replicates previously published findings that panobinostat is an effective targeted agent against DIPG tumor cells *in vitro* and in short-term *in vivo* efficacy studies, however a survival benefit for H3.3-K27M-mutant tumor-bearing mice was not evident, likely due to toxicity that necessitated dose de-escalation. Combination therapies that enhance the efficacy of panobinostat at less toxic doses may facilitate further clinical development of this therapy for DIPG patients.

## Supporting Information

S1 Fig Panobinostat treatment increases H3 Acetylation in human DIPG cell models. Full western blots corresponding to Fig 3D. Human glioma cell models were treated with either 0, 0.1, or 0.5 μM panobinostat for 48 h and then harvested for histone extraction. Histone lysates were separated via SDS-PAGE and blotted for H3 Acetylation (top panels) and total H4 (bottom panels). Arrows indicate the bands representing H3 Acetylation and H4. Western blot 1 and Western blot 2 correspond to the left-hand and right-hand blots, respectively, in Fig 3D.
(TIF)

S2 Fig No overall survival benefit with panobinostat treatments *in vivo*. **A.** Mice with tumors driven by PDGF-B and p53 loss harboring the H3.3-K27M mutation were treated with 20 mg/kg panobinostat (n = 13) or vehicle (25% DMSO, 0.25x PBS, 5% glucose, n = 10) administered via intraperitoneal (i.p.) injection once per day once a week beginning 21 days post-brainstem injection and continuing until mice reached humane endpoints. The mice were monitored daily and sacrificed upon moribund condition (lethargy, enlarged head circumference, ataxia, and/or > 25% weight loss) (p = 0.1176, log rank test). **B.** NOD-SCID mice (3 weeks old) were orthotopically injected with HSJD-DIPG-007 cells (passage 69) into the brainstem via stereotactic coordinates. Starting on day 23 post-implantation, mice were either not treated or treated with panobinostat prepared in 10% DMSO diluted in PBS-10% hydroxypropyl-beta-cyclodextrin (HPBCD) via i.p. injection at 10 mg/kg 5 days on 2 days off and 5 days on for a total of 10 doses (*p*>0.05, log-rank test). Shaded areas under the curves indicate treatment duration. **C.** Histological analysis of orthotopic xenograft model described in **(B)**, injected with passage 31 tumor cells, including H&E staining and IHC for Human Nuclei, H3K27me3, and Ki67 (20x objective, scale bars = 20 μm).
(TIF)
