# Supplementary figures and images for: Pre-Clinical Study of Panobinostat in Xenograft and Genetically Engineered Murine Diffuse Intrinsic Pontine Glioma Models
Source: PLoS One. 2017 Jan 4;12(1):e0169485. doi: 10.1371/journal.pone.0169485 (PMC5215670; doi:10.1371/journal.pone.0169485)

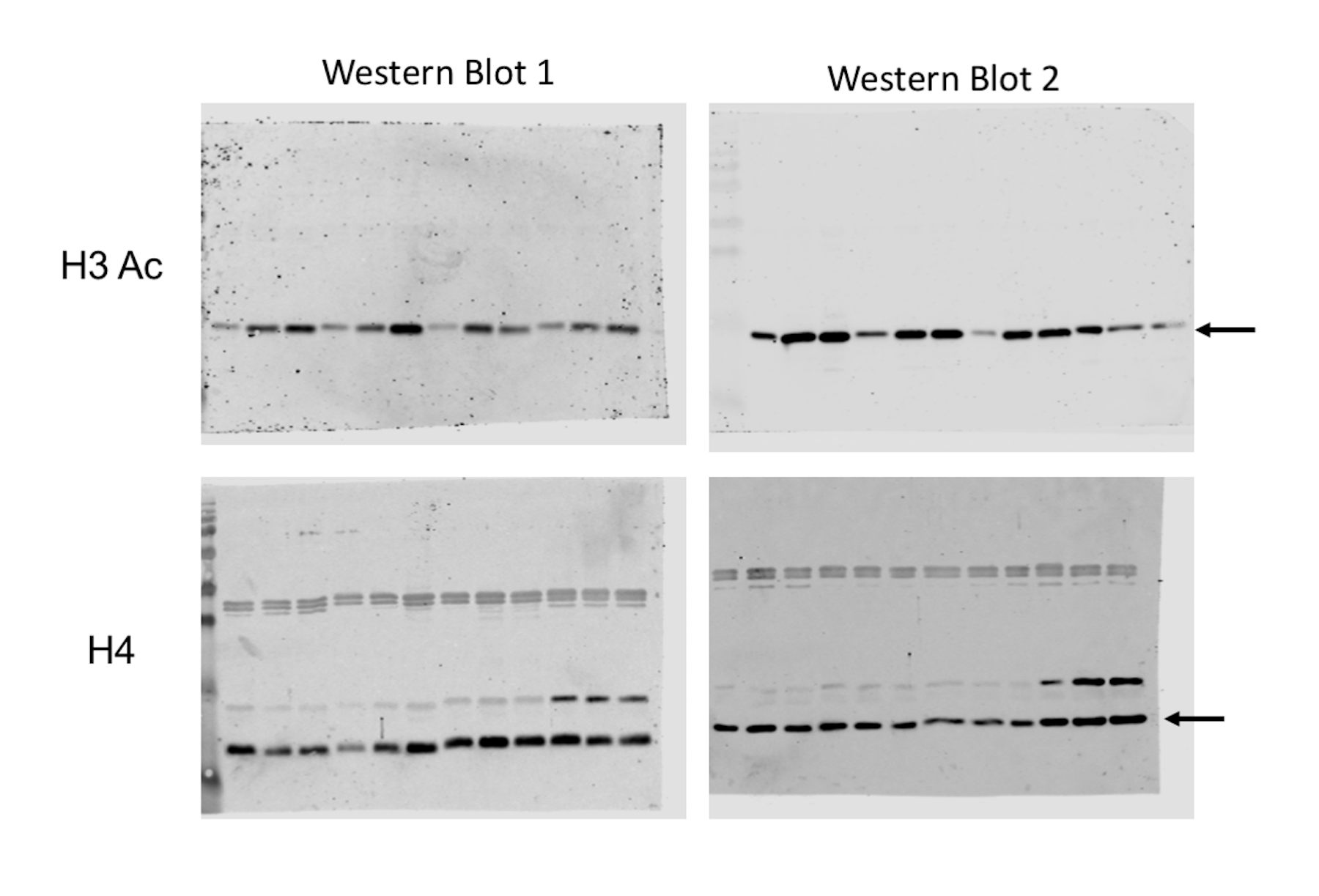

Supplement: S1 Fig — Full western blots corresponding to Fig 3D. Human glioma cell models were treated with either 0, 0.1, or 0.5 μM panobinostat for 48 h and then harvested for histone extraction. Histone lysates were separated via SDS-PAGE and blotted for H3 Acetylation (top panels) and total H4 (bottom panels). Arrows indicate the bands representing H3 Acetylation and H4. Western blot 1 and Western blot 2 correspond to the left-hand and right-hand blots, respectively, in Fig 3D. (TIF) [file pone.0169485.s001.tif]

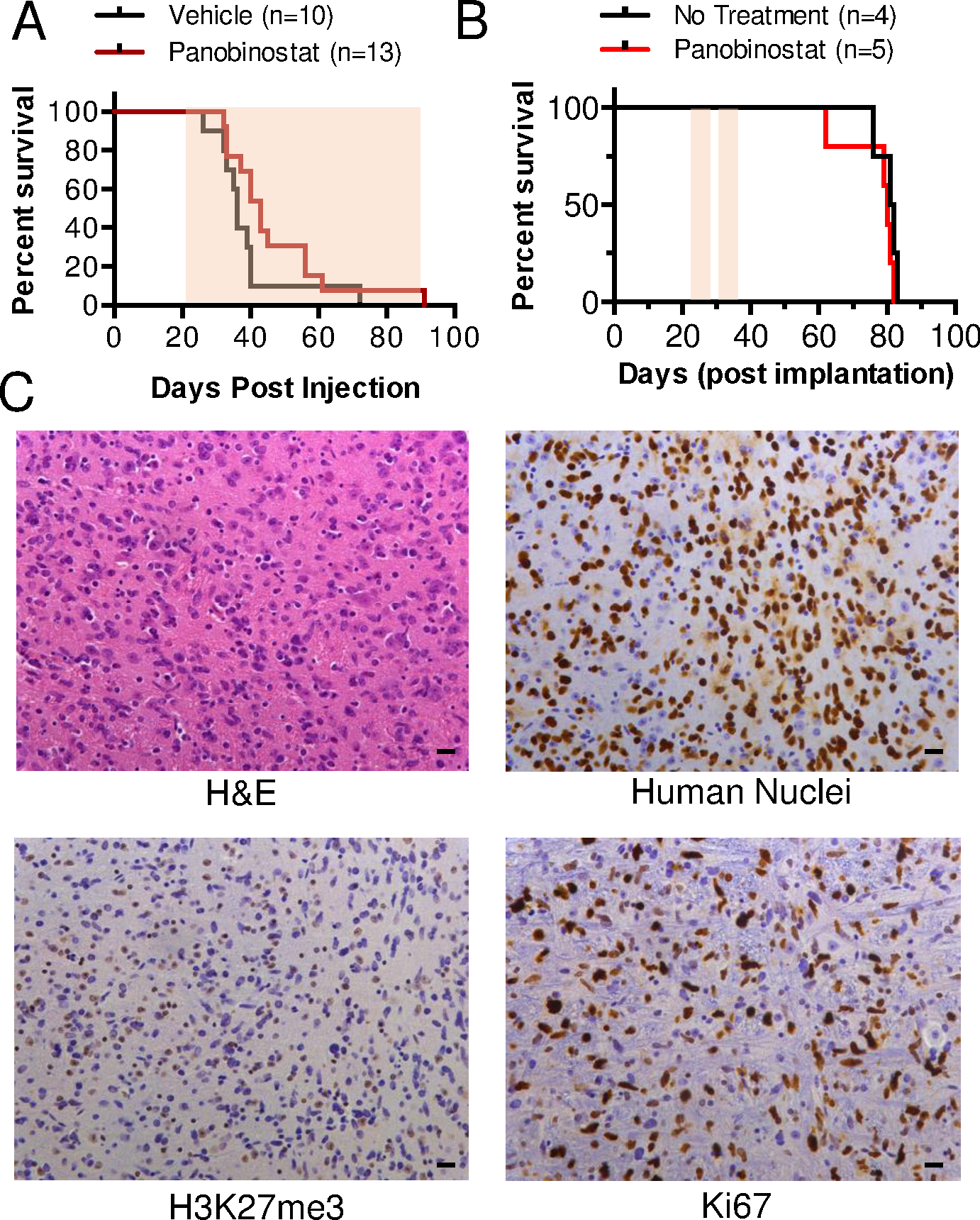

Supplement: S2 Fig — A. Mice with tumors driven by PDGF-B and p53 loss harboring the H3.3-K27M mutation were treated with 20 mg/kg panobinostat (n = 13) or vehicle (25% DMSO, 0.25x PBS, 5% glucose, n = 10) administered via intraperitoneal (i.p.) injection once per day once a week beginning 21 days post-brainstem injection and continuing until mice reached humane endpoints. The mice were monitored daily and sacrificed upon moribund condition (lethargy, enlarged head circumference, ataxia, and/or > 25% weight loss) (p = 0.1176, log rank test). B. NOD-SCID mice (3 weeks old) were orthotopically injected with HSJD-DIPG-007 cells (passage 69) into the brainstem via stereotactic coordinates. Starting on day 23 post-implantation, mice were either not treated or treated with panobinostat prepared in 10% DMSO diluted in PBS-10% hydroxypropyl-beta-cyclodextrin (HPBCD) via i.p. injection at 10 mg/kg 5 days on 2 days off and 5 days on for a total of 10 doses (p>0.05, log-rank test). Shaded areas under the curves indicate treatment duration. C. Histological analysis of orthotopic xenograft model described in (B), injected with passage 31 tumor cells, including H&E staining and IHC for Human Nuclei, H3K27me3, and Ki67 (20x objective, scale bars = 20 μm). (TIF) [file pone.0169485.s002.tif]
